# Biochemical Correlation of Thyroid Dysfunction Among Patients Presenting With Polymorphic Light Eruption

**DOI:** 10.7759/cureus.22944

**Published:** 2022-03-08

**Authors:** Roshni Mishra, Hemant Talanikar, Aishwarya Raheja, Mahendra Singh Deora, Siddharth Yadav, Divya Poulose

**Affiliations:** 1 Department of Dermatology, Dr D.Y. Patil Medical College Hospital and Research Center, Pune, IND; 2 Orthopaedics, Dr D.Y. Patil Medical College Hospital and Research Center, Pune, IND

**Keywords:** thyroxine, thyroid-stimulating hormone, sunlight, thyroid function tests, polymorphic light eruption

## Abstract

Objective

The pathophysiology of polymorphic light eruption (PMLE) is uncertain; however, it is considered to commonly involve an autoimmunological mechanism. It is a common condition, usually affecting subjects staying at temperate latitudes, and presents with eruptions post-exposure to sunlight and artificial UVR (ultraviolet radiation), lasting from hours to, in rare cases, days of exposure. This present study aims to compare biochemical thyroid function tests in cases of PMLE.

Methodology

The present case-control study was conducted with a total of 120 participants. Patients with polymorphic light eruption aged 18 years or above of either sex attending the dermatology outpatient department were included in the study. TSH (thyroid-stimulating hormone), T3 (triiodothyronine), and T4 (thyroxine) were analyzed among the participants. The data was recorded on a Microsoft Excel spreadsheet and analyzed using SPSS Statistics v. 21 (IBM Corp., Armonk, NY). The qualitative data was assessed in the form of numbers and percentages and the quantitative data was assessed using measures of central tendency such as mean and standard deviation. A chi-square test was applied to find out the association and their strength between the variables to validate the findings of the study. A p-value <0.05 was considered to be statistically significant.

Results

The TSH was elevated in 56 (93.3%) cases and two (3.3%) among the controls; T3 and T4 were low in 24 (40%) cases, and in seven (11.7%) among the controls.

Conclusion

PMLE usually has an autoimmune basis for its occurrence; similarly, thyroid disorders being themselves autoimmune in origin might lead to hypersensitivity reactions and generation of autoantibodies. We suggest that screening for thyroid should be conducted for all PMLE patients as they are at higher risk of developing thyroid disorders. The relationship between the two should be studied with a much larger cohort of participants to evaluate whether this is autoimmune-related or accidentally related.

## Introduction

Polymorphic light eruption (PMLE) is a photodermatosis whose pathogenesis is commonly linked to autoimmune mechanisms. It is a common condition, usually affecting residents of temperate latitude. It presents with eruption post-exposure to sunlight and artificial UVR (ultraviolet radiation), lasting from hours to rarely days of exposure. The eruptions are usually symmetrical, ranging from skin-colored to erythematous, varying from papules to pustules, and from vesicles to bullae in form. It commonly occurs over the sun-exposed sites of the body. Women are more commonly affected than men, and this condition usually manifests as a delayed-type hypersensitivity reaction against endogenous cutaneous photo-antigens [1].

It also has a genetic association as well, with family members of patients having similar chief complaints. The polymorphic light eruption can be produced by UVR from sunlight or artificial sources including sunbeds [2]. PMLE is a very common photodermatosis; the percentage of cases in western Europe and the USA is almost 10-20%. Though not a severe dermatologic condition, it usually interferes with day-to-day activities, mostly affecting outdoor workers. It belongs to the class of unknown photodermatoses, as the main pathomechanism behind its occurrence still remains unclear. It can affect healthy individuals on exposure to sunlight or artificial light, usually, there is no history or intake of photosensitizing agents. The main pathomechanism still remains unclear, but many theories speculate about its possible autoimmune origins [3].

In dermatoses such as melasma and polymorphic light eruption which are sunlight-associated, thyroid function disorders have been noted [3-5]. Herein we describe a comparative study of biochemical evaluation of thyroid function test carried out among 60 cases of PMLE with an equal number of controls.

## Materials and methods

The present case-control study was conducted in the department of dermatology, venereology, and leprology, Dr D.Y. Patil, Medical College, Hospital and Research Centre, Pimpri, Pune. Based on the inclusion-exclusion criteria detailed below, patients were divided into cases and controls with 60 patients in each group and a comparative study of biochemical evaluation of thyroid function test was carried out among all the participants.

The inclusion criteria were patients of polymorphic light eruption aged 18 years or above of either sex attending the dermatology outpatients department. The exclusion criteria were patients with lupus erythematosus, inflammatory photodermatoses, patients taking systemic steroids or using topical steroids in the previous three months, pregnant patients, patients on medication that cause photosensitivity (eg. quinolones, tetracyclines, sulfonamides, antimalarials, phenothiazines), patients taking specific treatment for PMLE in previous three months, like broad-spectrum sunscreens, hydroxychloroquine, omega-3 fatty acids, azathioprine, cyclosporine, and thalidomide.

The present study obtained approval from the institutional ethics committee of this institution. Written and informed consent was obtained from all patients. A detailed history regarding demography, occupation, age at onset, personal or family history of atopy, site of initial lesions, extent, duration of disease, seasonal variation, aggravating factors, type of footwear, household or occupational exposure to various materials was recorded. A dermatological examination was carried out and the morphology site size and distribution of lesions were noted. Subjects were asked about other chief complaints like itching, burning, or pain along with edema, oozing, or rise in temperature. The month of onset of rash, seasonal variation, treatment taken, discoloration was recorded as well. Other symptoms pertaining to thyroid disorder such as irritability, pain aches, constipation, diarrhea, sweating, heat or cold intolerance, dyspnoea on exertion, hoarseness of voice, eyesight changes, were noted. Similarly, the clinical history of the family and patients were recorded. The palpable thyroid gland was graded according to the five-point scale designed by Lewinski in 2002 [6]. Mucocutaneous examination in PMLE cases was done according to the six-point grading system designed by Fitzpatrick [7]. Distribution and type of lesions were also recorded. Whenever there was difficulty in diagnosis the patients were asked to avoid sunlight exposure by covering the exposed area for 15 days. A blood sample (5 ml) was collected from all participants for the estimation of serum thyroid-stimulating hormone (TSH) levels. Serum TSH (0.5 -5.0 micro IU/ml, normal range) level was found abnormal, further investigations for the estimation of serum triiodothyronine (T3) and thyroxine (T4) was done by immunotech kit.

Statistical analysis was done on a Microsoft Excel spreadsheet and analyzed using IBM SPSS Statistics, v. 21 (IBM Corp., Armonk, NY). The qualitative data was assessed in the form of numbers and percentages and the quantitative data was assessed using measures of central tendency such as mean and standard deviation. A chi-square test was applied to find out the association and their strength between the variables to validate the findings of the study. P-value <0.05 was considered statistically significant.

## Results

Out of 120 patients, 55 (45.8%) were male and 65 (54.2%) female. Their ages ranged from 25 - 65 years (as most of the population of this age group is exposed to daily sunlight exposure due to occupational reasons) with a median age of 42 years. There were two groups i.e., Case (Group 1) and Control (Group 2) of 60 patients each. The demographic details of the participants of the two groups are shown in Table 1. Their occupations included drivers (10); housewives (23); security (15); teachers (7); and farmers (65). However, the distribution of different occupations within the respective groups is shown in Table 1. The associated illness i.e., asthma, diabetes, hypertension, PCOS, and thyroid disorder among the groups are shown in Table 1. Thyroid function tests among the two groups are shown in table 1. The TSH was elevated in 56 (93.3%) cases in Group 1 and two (3.3%) in Group 2. The p-value for TSH, T3, and T4 was less than 0.005, hence the test was significant. T3 and T4 were low in 24 (40%) cases in Group 1 and seven (11.7%) cases in Group 2. There was the presence of hypothyroidism among 40% of cases and 11.7% of controls.

**Table 1:** Demographic details and biochemical parameters among the participants

		Group 1	Group 2	p-value
Sex	Male/Female	28/32	27/33	0.788
Occupation	Driver	3	7	0.073
	Farmer	31	34	
	Housewife	12	11	
	Security	12	3	
	Teacher	2	5	
Associated Illness	Asthma	0	4	0.001
	Diabetes	7	8	
	Hypertension	11	6	
	PCOS	1	5	
	Thyroid disorders	34	17	
	None	7	20	
TSH	Low	1	7	0
	Normal	3	51	
	High	56	2	
T3	Low	24	7	0
	Normal	36	44	
	High	0	9	
T4	Low	24	7	0
	Normal	36	49	
	High	0	4	

## Discussion

In our study, hypothyroidism was found in nearly 40% cases and 11.7% controls, and raised TSH level was found in 93.3% cases and 3.3% controls. In a study by Seetharam et al., [4] nearly 25.9% cases were hypothyroid along with polymorphic light eruption, and only 7.5% of controls were hypothyroid. Sharma et al., [3] in 2014 found out that nearly 18% cases and 5% of controls were found to have overt hypothyroidism along with subclinical hypothyroidism in 6% cases and 1% controls. Hasan et al., [5] found out that polymorphic light eruption had an autoimmune basis for origin and females showed a higher number of cases compared to males. The study conducted by Kochupillai showed a similar relationship between polymorphic light eruption and thyroid, proving that it was not an accidental finding [8].

**Figure 1:**
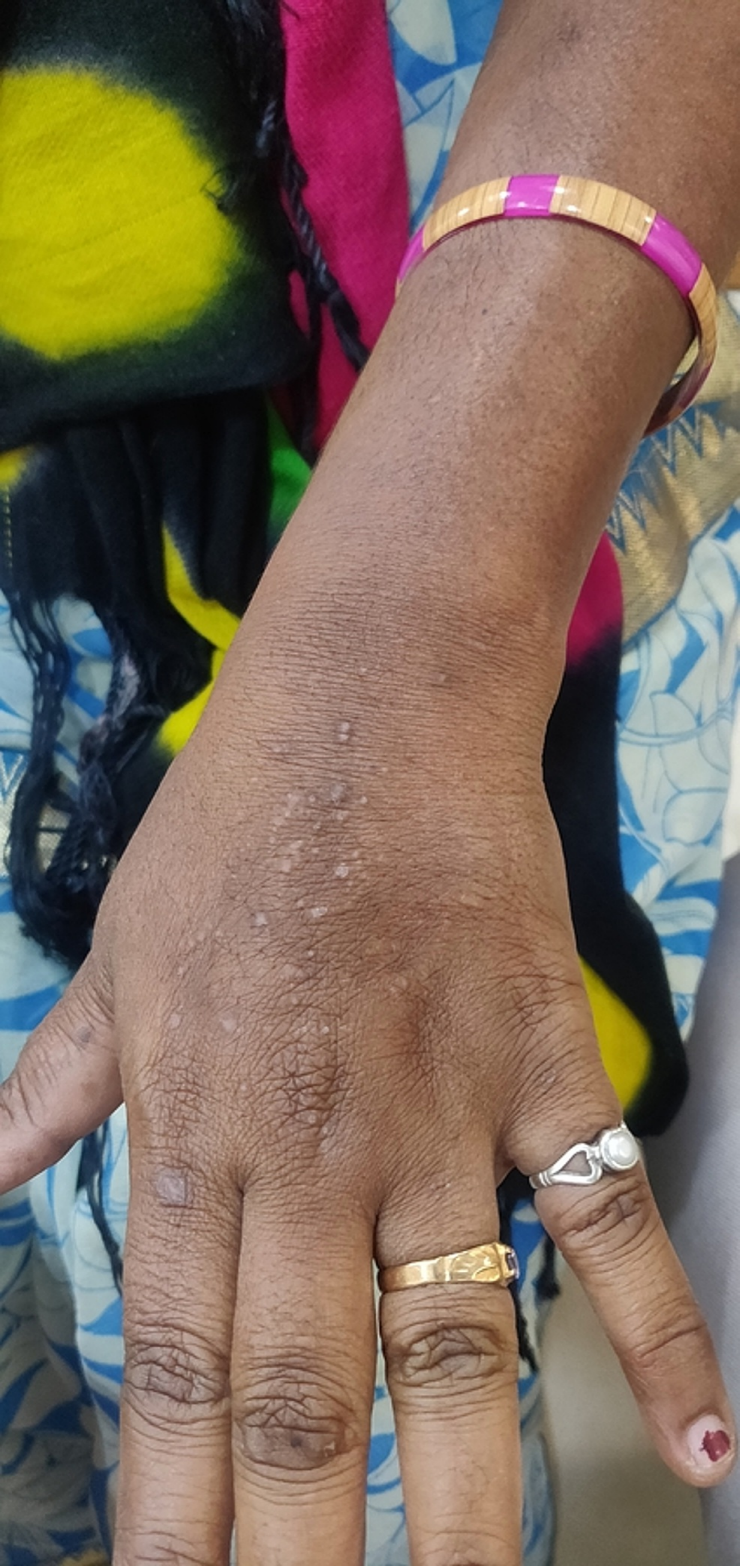
Polymorphic light eruption lesions over the dorsum of the hand. Multiple small papules present over the anterior aspect of the hand, associated with itching sensations, and were seen on exposure to sunlight.

**Figure 2:**
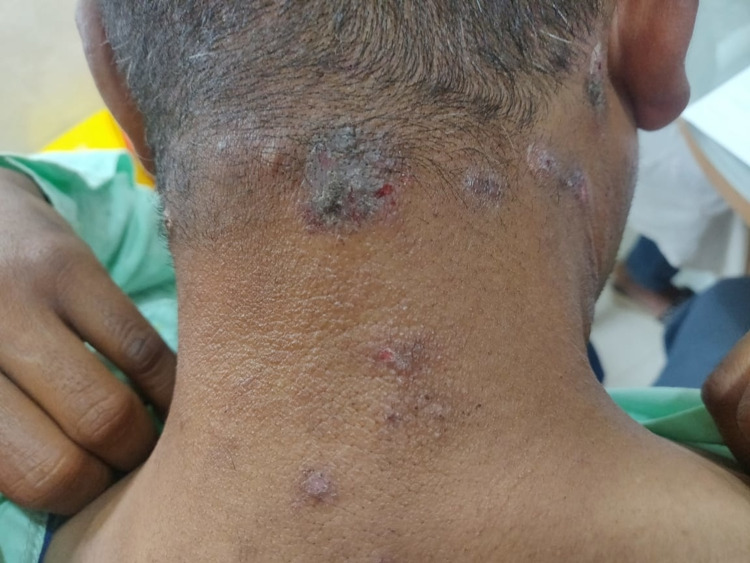
Polymorphic light eruption over the nape of the neck. Note the multiple hyperkeratotic plaques present over the nape of the neck.

Females show a slightly higher preponderance than males and in our study (54.2%) were females and (45.8%) were male. The prevalence was found to be 10% in the United States [9], 0.56% in India [10], 15% in the United Kingdom [4,9], and 5% in Australia [4,10]. In the UK, 15% of the affected population were females [10]. The disease was more common among farmers due to extensive outdoor activities. In various studies, the increased incidence was seen in housewives usually due to household activities that involved exposure to sunlight in the maximum number of cases [10-12]. In our study, the disease was more active among farmers (51.7%) than followed by the housewives. Polymorphic light eruption has a delayed onset, and an abnormal reaction to sunlight that subsides without scarring. As it has various morphological types, it is referred to as polymorphic. It starts mostly before 30 years of age. Some Fitzpatrick skin types are more susceptible to developing PMLE, with the highest prevalence in people with skin type I and lowest in skin type IV or greater [13]. Usually, the most commonly affected sites were the sun-exposed area which included the anterior aspect of legs and hands (Figure 1), face, nape of the neck (Figure 2), anterior aspect of legs and hands, trunk, nose, scalp upper chest as these sites had maximum exposure to sunlight. The lesions were grouped vesicles, papules, or plaques depending upon the chronicity of the disease. They were symmetrically distributed and were also associated with discharge in a few cases.

Polymorphic light eruption is a delayed-type of hypersensitivity response to a UV-induced allergen (photoallergen), usually proved by the histopathological findings that show: (1) the infiltrating cells are mainly T-lymphocytes mostly CD4+ in the early lesions and CD8+ later; (2) Langerhans cells and dermal macrophages are also seen [14].

In our study, the percentage of association of various disorders among the cases were as follows: 56.7% thyroid disorders, diabetes 11.7%, and PCOS 1.7%. Due to the high number of associations with thyroid disorders, the thyroid function tests consisting of TSH level, T3 level, T4 level, were also sent for analysis. A greater number of PMLE patients are required to signify the relationship between the two, to establish it as an autoimmune association or accidental relationship. 

## Conclusions

PMLE usually has an autoimmune basis for its occurrence; similarly, thyroid disorders being themselves autoimmune in origin might lead to hypersensitivity reaction and generation of autoantibodies. Thyroid disorders are usually associated with xerotic skin; due to constant dryness and loss of epidermis, the skin is directly exposed to sunlight which might aggravate the formation of polymorphic light eruption lesions over the exposed part.

We suggest that screening for thyroid should be done for all PMLE patients as they are at higher risk of developing thyroid disorders. The relationship between the two should be studied with a much higher number of cases to evaluate whether this condition is autoimmune-related or accidentally related.
